# A Technical Feasibility Study of the Recovery of Used Lubricant Oil Using Ceramic Ultrafiltration Membranes

**DOI:** 10.3390/membranes16050164

**Published:** 2026-05-01

**Authors:** Madina Mohamed, Pieter Vandezande, Anita Buekenhoudt

**Affiliations:** Flemish Institute for Technological Research NV (VITO), Materials and Chemistry (MATCH), Boeretang 200, B-2400 Mol, Belgium

**Keywords:** used lubricant oil, ceramic membrane, membrane grafting, membrane flux

## Abstract

This laboratory-scale experimental study investigated the purification level of used lubricant oil (ULO) filtration using a large variety of ceramic UF membranes, allowing for treatment at high temperatures unreachable for polymeric membranes. Varying pore sizes (5 nm, 10 nm, 30 nm, and 100 nm) were included as well as a range of materials (Al_2_O_3_, TiO_2_, and ZrO_2_). Moreover, four different grafting techniques were applied to alter the surface chemistry of the native membranes from hydrophilic to more hydrophobic or oleophilic, intending to further increase UF flux and/or retention. Benchmark native 10 nm TiO_2_ membranes shows a stable flux of 7 to 9 kg/h·m^2^ at 110 °C, strong (metal) impurity removal, and unexpected high water retention. All other membranes tested show fluxes that never exceed the ones for the 10 nm benchmark membranes, elucidating that surface chemistry does not help to improve the flux. In general, membrane performance is very similar for all membranes, except for flux and water retention. Systematically, high-flux membranes show high water retention, while very-low-flux membranes preferentially pass water. The variation in flux and water retention as a function of membrane pore size (before grafting) shows that surface chemistry only plays a role when the effective pore size becomes small. The study results allow for the selection of the best membranes for initial ULO treatment.

## 1. Introduction

Lubricating oil is a petroleum-derived product designed to reduce friction in machinery, minimize wear on moving parts, and improve the overall efficiency of automotive engines and industrial machinery. Virgin lubricating oil typically contains 80–90% so-called base oil and 10–20% chemical additives. These additives include surfactants such as antioxidants, detergents, and viscosity enhancers, as well as rust and wear inhibitors [1,2].

Throughout the process of using lubricant oil, the chemistry of lubricating oil undergoes changes due to temperature buildup, resulting in additives breaking down, causing corrosion and oxidation. Simultaneously, the level of impurities increases with the change in the viscosity, acidity, flash point, and metal content of the oil, leading to inefficient performance of the relevant system, a shortened lifespan of the equipment, and premature failures. So, following usage, the oil’s quality deteriorates, causing the loss of its essential properties, rendering it ineffective and compromising its ability to protect and lubricate the system [3,4,5,6].

As a consequence, over time, the oil accumulates wear and oxidation impurities, including sludge, oxidation byproducts, carbon particles, and ash. These contaminants often contain various metal elements, originating not only from wear and fouling but also from the lubricant additives themselves [1,2]. Moreover, during use, the lubricant oil also gets contaminated with % levels of water, diesel and naphtha. Diesel and naphtha typically consist of C3 to C20 hydrocarbons, while base oil is composed of longer hydrocarbons (C25 to C40). The viscosity of the used oil is typically very high and quantified by its kinetic viscosity in the range of 50 to 60 cStokes at 40 °C.

The environmental disposal of used lubricating oil (ULO) poses a hazard to soil, water, and air, given its classification as a toxic and non-biodegradable waste [6,7]. Due to the high management costs of ULO, improper enforcement of environmental regulations, and mismanagement of its disposal, waste lubricant oil was commonly dumped into water bodies, causing an increase in chemical oxygen demand (COD), adversely affecting aquatic life [8]. As a consequence, efficient ULO collection has now been organized. In 2017, approximately 1.6 Mt of ULO was collected across the EU, of which 1 Mt was sent for re-refining. This process yielded around 680 kt of recovered base oil, produced by 27 re-refining facilities [9].

Recently, 80% of the collected ULO underwent recycling for reuse or regeneration into new products. This change comes in response to the continuous growth in the annual production of used automobile lubricating oil [9]. Various recycling methods are employed to convert ULO into valuable fuel products or chemical feedstocks. In the EU region, ULO is commonly recycled into base oil, which is then reused in the formulation of new lubricants. Current recycling techniques mainly include distillation and/or solvent extraction, complemented by techniques such as acid/clay treatment, hydrogenation/clay treatment, and pyrolysis with microwave heating [9,10,11,12,13,14].

Membrane technology has gained significant attention for its potential to address environmental challenges, improve industrial processes, and contribute to sustainable development. With the increasing urgency to mitigate carbon emissions and control the impact of climate change, the development of advanced membrane technology capable of liquid treatment in the oil and gas industry holds immense significance [13,14,15,16,17,18,19,20,21,22,23,24,25]. Several studies on waste-oil treatment have also explored the use of ultrafiltration (UF) membranes for the purification of ULO. Widodo et al. [26] investigated the use of hydrophobic polypropylene hollow fiber membranes for ultrafiltration of pre-treated (particle removal using 5 µm filter) waste engine oils. Membrane performance was evaluated across pressures of 0.4–1.2 bar and temperatures of 30–60 °C, revealing that oil flux increased under higher pressure and temperature while impurity rejection remained relatively constant. Due to the high feed viscosity, flux values were quite low and ranged from 0.09 to 0.19 L m^−2^ h^−1^. However, the UF process effectively removed impurities from the waste oil, including ash (90–99%), water (78–82.5%), zinc (28.9–43.8%), and calcium (50.1–58.9%), thereby improving the treated oil’s viscosity, density, and color [26].

Cao et al. [27] tested three types of polymer hollow fiber membranes (polyethersulfone (PES), polyvinylidene fluoride (PVDF), and polyacrylonitrile) with different pore sizes to recycle ULO. They used ULO pre-treated by centrifugation. Fluxes at 40 °C were typically very low and ranged from 0.2 to 1.2 L/hm^2^. A UV–VIS absorbance rejection rate of 99.6% was observed with the PAN UF membrane, surpassing the rejection rates of microfiltration membranes made with PES and PVDF. Both the liquidity and flash point of the recovered lubricant oil were highly improved [27]. Nebesskaya et al. [28] synthesized a 23 nm ultrafiltration membrane using poly(acrylonitrile-co-methyl acrylate) (P(AN-co-MA) for the separation of used engine oil (UEO), strongly diluted with toluene (100 g/L). The membrane again achieved good rejection of major contaminants in used engine oil, including polymerization products and metals [28]. Due to toluene dilution, observed fluxes were higher here.

From the above results, it can be observed that UF leads to good first purification of ULO, but fluxes are typically low. This is caused by the high viscosity of the oil combined with the low temperatures used for filtration with polymeric membranes and the high level of impurities. To increase useful filtration temperatures and fluxes, the use of ceramic UF membranes was studied as well. The authors investigated the removal of ash and color contaminants from ULO by employing a porous tubular inorganic membrane, with pore sizes ranging from 0.05 µm to 0.2 µm, for initial filtration, followed by the use of an adsorbent to further eliminate color and odor from the oil permeate. The membrane filtration step was operated at elevated temperatures, typically between 80 and 185 °C, to reduce oil viscosity and enhance permeation. Across multiple examples, permeance values were reported between approximately 2 and 16 Lm^−2^h^−1^bar^−1^, depending on pore size and operating temperature. This demonstrates that ceramic ultrafiltration membranes can be effectively applied for waste-oil purification if operated under high temperatures to overcome viscosity-related flux limitations [29].

This paper aims to reveal if changing the surface chemistry of ceramic membranes can further improve the flux and/or removal efficiency. Native ceramic membranes, typically metal oxide membranes, are very hydrophilic, and thus increasing the hydrophobicity or oleophilicity of these membranes could have a beneficial effect on their performance in ULO removal. Therefore, this research investigates the usage of a wide variety of both native and grafted porous ultrafiltration ceramic membranes for the purification of real-life ULO samples from the oil and gas industry. The impact of membrane pore size and membrane surface modification on membrane flux and selectivity are examined. The research aims to contribute to the development of innovative ceramic membrane-based processes for waste-oil purification, addressing critical environmental challenges and promoting sustainable practices. Additionally, the research seeks to enhance carbon circularity by facilitating the reuse of recovered base oil in the production of new lubricants, while also working toward reducing the CO_2_ footprint.

## 2. Materials and Methods

### 2.1. Membranes and Grafting Techniques

A broad range of commercial UF ceramic membranes of 25 cm length (monochannel tubes with an outer diameter of 1 cm and an inner diameter of 0.7 cm) for liquid filtrations were obtained from the company Inopor, Veilsdorf, Germany (in the framework of the Horizon Europe project CUMERI). The membranes’ top layers varied in pore size (from 3 to 100 nm) and in material (TiO_2_, ZrO_2_, and Al_2_O_3_). Most of the membranes had an alumina support, but one full TiO_2_ membrane was also included in this study. An overview of the used membranes together with their molecular weight cut-offs (MWCOs) as shared by the supplier can be found in Table 1 (the membrane types are defined by the pore size and material of the top layer). The customized membranes were prepared in-house, using the native ceramic membrane supports mentioned above and chemically modifying them with different grafting technology. The ceramic supports offer wide chemical robustness and enable their use at elevated temperatures above 50 °C, the typical limiting temperature for polymeric membranes. The grafting technologies were selected to keep a similar chemical resistance and lead to chemically bonded grafted groups stable up to at least 150 °C. The temperature range up to 150 °C is particularly advantageous for treating viscous ULO feed streams.

**Figure 1:**
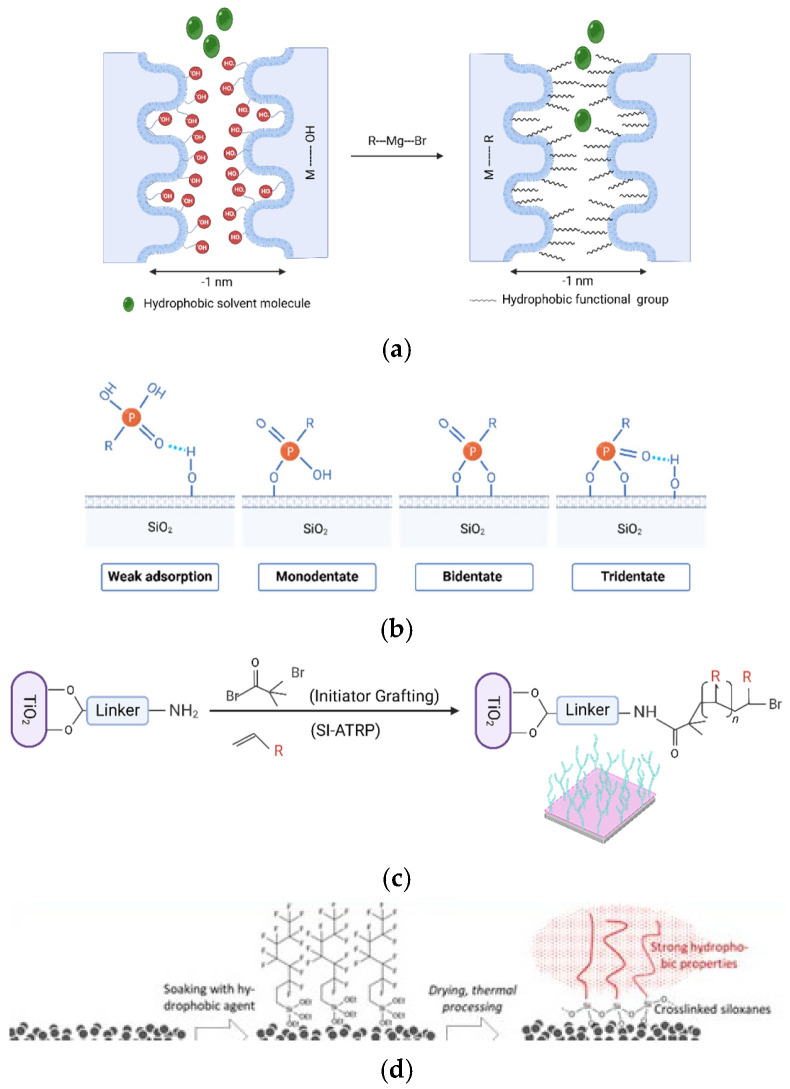
Illustration of ceramic membranes. (**a**) Grignard grafting, (**b**) phosphonic acid grafting, (**c**) surface-initiated atom transfer radical polymerization grafting, and (**d**) silanation. Adapted from [36,37,44].

The different native metal oxide membranes all had abundant -OH groups on their entire pore surface, making these membranes highly hydrophilic. By grafting different chemical groups on their surface, we intended to make these membranes more hydrophobic and/or more oleophilic, which is beneficial for oil filtration. Four different, previously developed chemical modification techniques were used for the modification of the surface chemistry of the membranes, namely: silanation (SI), Grignard grafting (GR), phosphonic acid grafting (PA), and surface-initiated atom transfer radical polymerization (Si-ATRP). All four techniques are schematically visualized in Figure 1. As can be observed, each grafting technique yields different surface and pore chemistry modifications. Details of the first 3 grafting techniques (SI, GR, and PA) and the characteristics of the resulting membranes can be found in the review paper [30]; a short overview is given here.

Silanation is one of the most well-known grafting techniques. It uses silane reagents to cause a condensation reaction with the -OH groups on metal oxides. High surface coverages are typically reached, but bond stability is dependent on the metal oxide and grafted group. Fraunhofer IKTS optimized this technique to create robust, strongly hydrophobic UF membranes that are also semi-commercially available at Inopor (HOC membranes) [31,32]. In these membranes, both the support and top layers are chemically modified. Particularly for this study, IKTS used the HOC reagent in 2 concentrations (1% and 2%). Moreover, they also prepared membranes with an extra hydrophilic pore surface (S-HIGS membranes). Quality control of silanated membranes was typically done by contact angle measurements and/or Liquid Entry Pressure determination [31,33].

Grignard grafting is the proprietary grafting technique that was developed by VITO in collaboration with the University of Antwerp and recently commercialized by the SME company A-membranes www.a-membranes.com (accessed on 20 January 2026). When Grignard reagents were applied, partial replacement of the native membrane’s -OH groups occurred, leading to the direct bonding of the intended organic groups to the metal atoms through robust M-C bonds, without the involvement of oxygen atoms (as shown in Figure 1). The reaction (as applied at VITO) is possible on TiO_2_ and ZrO_2_ but not on Al_2_O_3_, leading, in the majority of cases, to membranes with only chemically modified top layers and not supports. In this study, VITO used Grignard grafting to alter the surface chemistry of a variety of TiO_2_ and ZrO_2_ membranes to make them more hydrophobic (full hydrophobicity cannot be reached due to the partial surface coverage). The quality of the GR-grafted membranes was typically measured by contact angle measurements and/or water permeability decline after grafting [34,35,36].

Phosphonic acid grafting is known in the industry and involves a condensation reaction that forms one or more oxygen-bridged M-O-P-R bonds [30]. High surface coverage can be reached. A notable limitation of the phosphonic acid method is the limited range of functional groups commercially available and the limited stability of the formed bond in the case of Al_2_O_3_ (or SiO_2_, but that is not relevant for this work). In this study, VITO used the technique to create hydrophobicity on some of the TiO_2_ top layers; the alumina supports were not modified. Increased hydrophobicity was again evident from contact angle measurements and/or water permeability decline after grafting [34,35].

To enlarge the possible surface chemistries even further, a fourth grafting technique recently optimized by VITO for membrane modification was included in this research [37]. Surface-initiated atom transfer radical polymerization (Si-ATRP) offers a way to create small polymer chains on the entire pore surface of different membranes. The controlled approach enables the transfer of activated monomer units to grow well-defined polymer chains from the membrane surface. Si-ATRP provides advanced and flexible capabilities for designing robust, custom materials with a wide range of chemistries. The effective grafting of the intended polymer brushes was proven using IR measurements [38]. The quality of the grafted membranes was defined by flux decline during grafting, and flux and retention measurements were done with defined mixtures in DMF and toluene [39].

**Figure 2:**
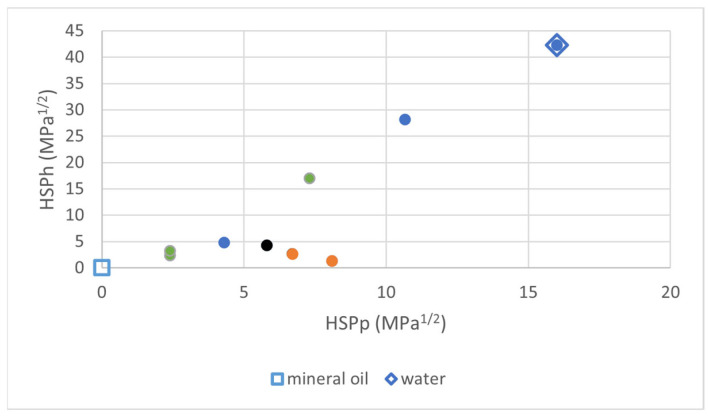
Plot of HSP values for all chemistries used within CUMERI. HSP_d_ is defined by different colors (see text; in rising order: blue, orange, green, and black). HSP values of mineral oil and water are added for comparison.

In this study, VITO used these techniques to produce a wide variety of hydrophobic and oleophilic top layers, starting from membranes with a relatively large pore size (10 and 30 nm). An overview of all the membranes (native and grafted) utilized in this study is shown in Table 2. In total, 35 different membranes were included. In the CUMERI project, and for this study, the Hansen Solubility Parameters (HSPs) [40] were used to quantify the affinity of the different membranes to water (hydrophilicity/hydrophobicity) and oil (oleophilicity/oleophobicity). HSPs were calculated in the following way: 1. for native membranes, the HSP values (close to water) derived in [41] were used; 2. for Grignard-grafted membranes, the method described in [41] was used, taking into account partial coverage of the surface by grafted groups and remaining OH groups; 3. for SI-ATRP-grafted membranes, HSP values of the grafted polymer were calculated using HSPiP software (version 6.1.02) [42]; 4. for the membranes silanated using long perfluoroalkyl silanes, the HSP values of polytetrafluoroethylene (PTFE) calculated by the HSPiP software were used. Figure 2 plots the polar and hydrogen-bonding HSP values for all membrane chemistry types prepared within the study. The color code indicates the values of the dispersion HSP (blue 14–16, orange 16–18, green 18–20, and black 20–22 MPa^1/2^). The figure shows that the membranes synthesized and tested in this study span a wide variety of surface chemistries, ranging from the very hydrophilic native membranes with a high HSPp and HSPh (top right corner of the plot) to very hydrophobic grafted membranes with a low HSPp and HSPh (bottom left corner of the plot). For comparison, we have also marked the water HSP values (open diamond) and the mineral oil HSP values (open square) [43], each positioned on one side of the plot. The oleophilicity of a membrane is defined by the 3D distance from its HSP point to the mineral oil HSP point.

### 2.2. Feed Solutions’ Characteristics and Properties

This research was performed in the framework of the EU project CUMERI (cumeri.eu), partnering with one of the bigger European ULO re-refiners, Osilub www.osilub.fr"(accessed on 5 February 2025). Osilub is currently re-refining ULO collected by Veolia using a 3-step distillation process, leading to clean base-oil products that are reused by Total Energies to make new lubricants. The ULO processed by Osilub is a mixture sourced primarily from trucks, with approximately 90% obtained from cars and 10% from industrial applications. Their energy-intensive re-refining process consists of a first distillation to remove water and naphtha, a second distillation to remove the diesel, and the final third distillation to remove all other impurities. Before going into the distillation train at Osilub, the ULO is heated up to 90 °C and neutralized by NaOH addition, allowing for better purification and helping with color removal.

In this study, several oily feed streams sourced from OSILUB were tested in the UF experiments: ULO as collected without neutralization, ULO after neutralization and first distillation (ULO 1st dist), ULO after neutralization and second distillation (ULO 2nd dist), and OSIL150, one of the clean base-oil products of Osilub. We remark that it was not possible to obtain an ULO stream directly after neutralization. Table 3 gives an overview of the most important characteristics of the different feed streams. It quantifies water content, kinetic viscosity at 40 and 100 °C, acidity number (TAN), level of oxidation and nitration components (measured by UV/VIS absorption), and diesel and naphtha content. For comparison, the characteristics of a commercially available clean lubricant oil (LO) were included in the table (mineral oil 15W-40). The measured water content reflects two distinct types: water dispersed within the oil phase, stabilized by the surface-active properties of the additives present in the ULO, and free, unbound water, which is not stabilized and can pass through the membrane.

**Figure 3:**
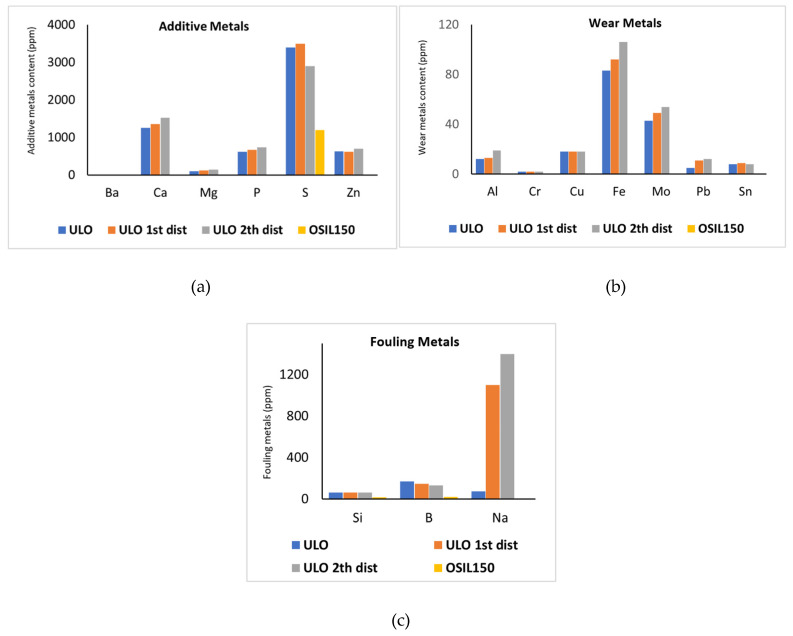
Metal content (ppm) of different ULOs. If no bar is visible, the content of that particular metal is zero. (**a**) additive metano content neededls. (**b**) wear metano content neededls. (**c**) fouling metano content neededls.

The metal content of the different oily feeds of Osilub is visualized in Figure 3. The metals are divided into metals coming from the additives, from fouling (like Si from sand), and from wear. The various treatment steps of the ULO obviously influence the characteristics of the oil streams. The intended effect of the different distillation steps is visible: water and naphtha removal by the 1st distillation, diesel removal by the 2nd distillation, and removal of almost all metal impurities by the 3rd distillation. Also, notice the increasing content of Na after the distillation steps, which actually originates from the NaOH neutralization step before. Also, remark that for the Osilub product, OSIL150, some S, P and Si is still present in the further clean base oil. The relatively high S level categorizes this as a Type 1 oil. To remove all S and get a Type II oil, an extra treatment like a hydrogenation step would be needed.

### 2.3. UF Tests

**Figure 4:**
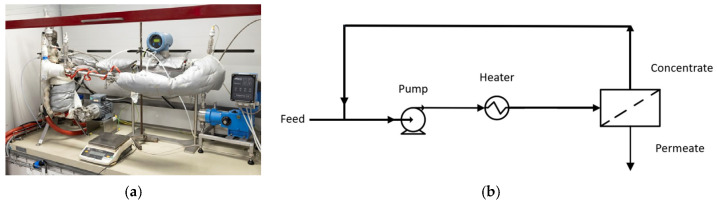
(**a**) Picture and (**b**) schematic representation of the membrane filtration system.

Batch filtration was used for all UF experiments. The UF tests were performed at VITO using a home-made, cross-flow filtration loop. The set-up consisted of a 1 L feed tank connected to a high-pressure pump that delivered the feed solution across the active surface of the membrane. The permeate was collected downstream of the membrane, while the retentate was recirculated back to the feed tank. Flow rates and pressure were fully adjustable to evaluate membrane performance under a variety of operational conditions. The system was instrumented with temperature control, ensuring stable thermal conditions throughout the experiment. To allow for working at high temperatures, the full loop was isolated. Figure 4 shows a scheme of the filtration loop and a picture of the isolated system.

Unless mentioned otherwise, a fixed procedure was used for UF experiments (1 L of oily feed was introduced into the feed tank and subsequently circulated at 2 m/s and heated up to 110 °C). This typically took 1 to 2 h. Once the temperature stabilized, a 100 mL feed sample was collected. The pressure was then raised to 10 bar, and a 100 mL permeate sample was collected, aiming for 11% volume recovery. It was checked that the performance of the membrane was the same when heating was done with the membranes in by-pass. Flux and operational conditions were continuously monitored throughout the process. Finally, a 100 mL retentate sample was collected, and all samples were analyzed after testing. All analysis was performed by an external company (Alpha Maintenance Systems, Belgium), specialized in the analysis of these streams (mainly following methods standardized in the field and mentioned in the next sentence). The parameters analyzed were: water content (in ppm, ASTM D6304C) [45], kinetic viscosity at 40 °C and/or 100 °C (in cStokes, ASTM D7279A) [46], total acidity number TAN (in mg KOH/g), ASTM D8045), oxidation and nitration level (Abs/0.1 cm, ASTM E2412) [47], diesel and naphtha (% measured by gc), and different metals (in ppm, ASTM D5185) [48].

## 3. Results and Discussion

### 3.1. Performance of Benchmark 10 nm Native Membranes

**Figure 5:**
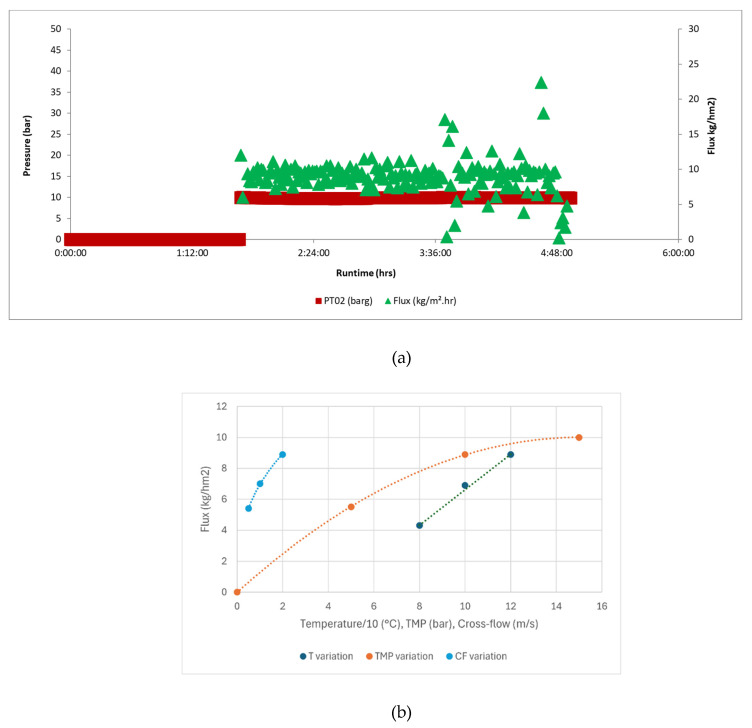
(**a**) Evolution of the flux of a benchmark 10 nm membrane with time in standard conditions (**b**) and variation in the stable flux with different parameters. T variation was performed at 10 bar and 2 m/s, TMP variation at 120 °C and 2 m/s, and CF variation at 120 °C and 10 bar.

From the whole series of membranes used in this study, we decided to use the 10 nm TiO_2_ native membranes as the benchmark membranes. A fresh 10 nm native membrane was first used to filter as-received ULO (ULO without neutralization) in our standard conditions (see Section 2.3). As soon as the temperature was stable and the pressure was applied, the membrane exhibited a relatively small but stable UF flux of 9 kg/h·m^2^ at 110 °C, as shown in Figure 5a. We noticed that this flux value varied somewhat depending on the batch of ULO received, ranging from 7 to 9 kg/h·m^2^. To get an idea of the factors influencing this flux, we investigated the effect of different parameters: temperature (T), transmembrane pressure (TMP), and cross-flow (CF). Figure 5b shows the results. The strong effect of temperature follows the large variation in the permeate dynamic viscosity (calculated with the calculation tool Olezol.com based on the measured kinetic viscosities of the permeate at 40 and 100 °C and the oil density at the measurement temperature). The variation with cross-flow follows a power law with an exponential of 0.36, close to one-third of that expected for laminar flow of viscous oil streams. The variation in TMP (polynomial fit) highlights the existence of a limiting flux around 15 bar and points to the existence of a concentration polarization layer or a cake layer created by the retained impurities in the retentate. To assess the strength of this impurity layer, we also determined the flux of impurity-free base oil (see Section 3.2).

**Figure 6:**
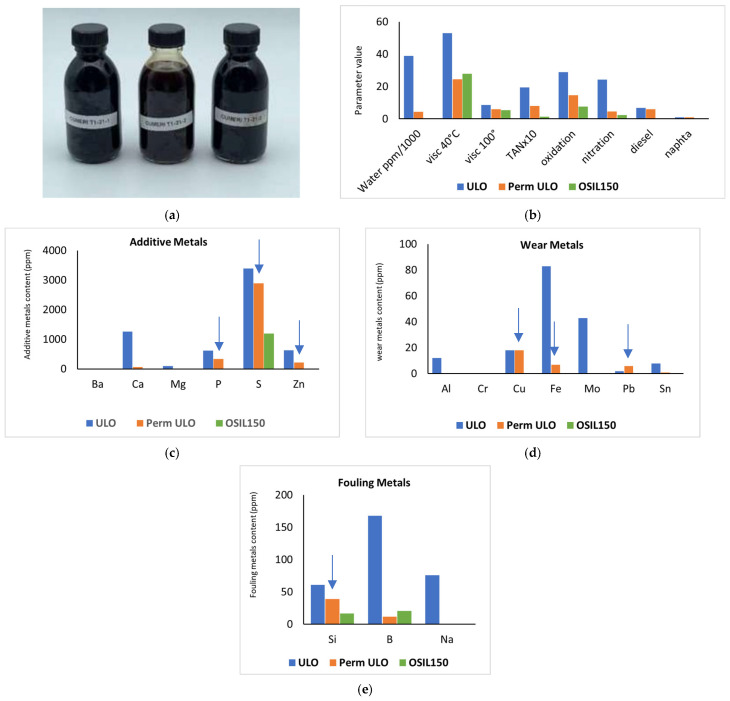
Performance of 10 nm native membrane in as-received ULO. (**a**) The picture shows the feed, permeate, and retentate from left to right. Graph (**b**) shows the values for general characteristics (parameter on the *x*-axis, water content in ppm, kinetic viscosity at 40 °C and/or 100 °C in cStokes, total acidity number TAN in mg KOH/g, oxidation and nitration level in Abs/0.1 cm, and diesel and naphtha in %). Graphs (**c**–**e**) show metal content in ppm for additive metals (left), fouling metals (middle), and wear metals (right). If no bar is visible, the value of the related parameter is zero.

Figure 6 illustrates the quality of the permeate created: it visualizes the characteristics of the feed (blue), the permeate (orange) and the re-refined product from OSILUB, OSIL150 (green). Note that the permeate from this filtration remains black, similar to the feed and retentate. Figure 6 shows that the water removal was unexpectedly high, achieving levels near or above 90%, and was most likely caused by the above-mentioned stabilization of the water with surfactant-like additives or other impurities in the oil. Viscosity, total acid number (TAN), oxidation, and nitration levels were reduced by 50 to 70%. As expected for UF, there was no removal of diesel or naphtha. Also, strong metal removal was observed up to the target levels, with the exception of phosphorus (P), sulfur (S), zinc (Zn), silicon (Si), copper (Cu), iron (Fe), and lead (Pb), indicated by arrows. We remark here briefly that the permeate quality remained consistent regardless of the used temperature, TMP and cross-flow rates (1 to 4 m/s). Moreover, the performance (flux and retention) was also the same when the membrane was reused after being kept in xylene in between the tests. This means that xylene is a good cleaning agent for the Osilub type of ULO (as also communicated by the company).

**Figure 7:**
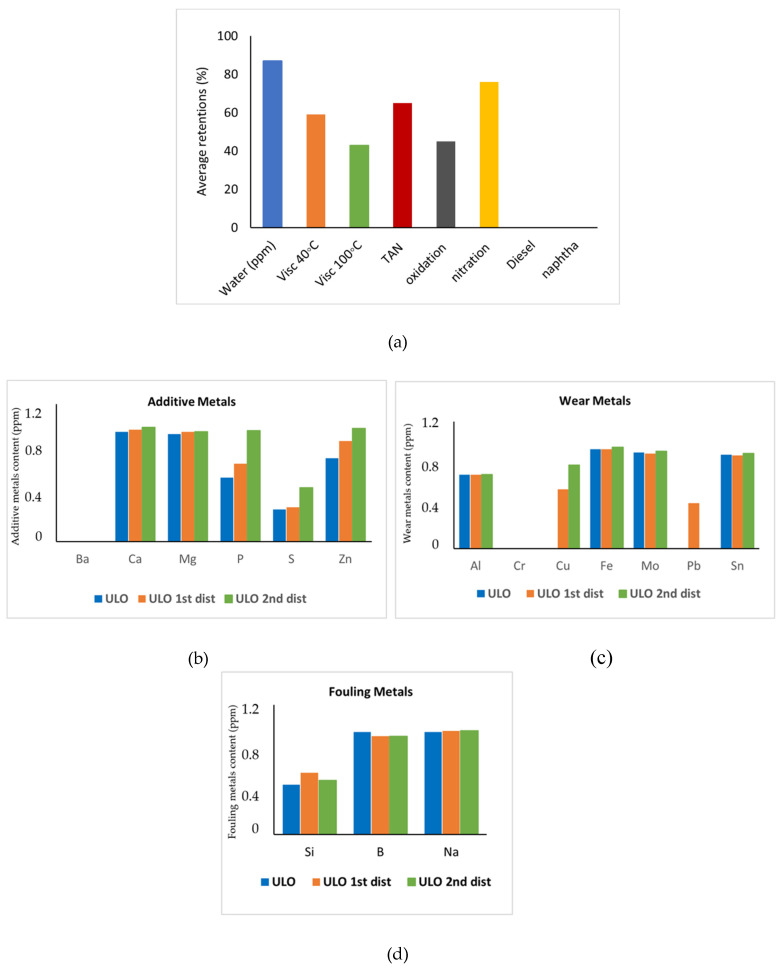
(**a**) Retentions of 10 nm native membranes for ULO with different pre-treatments. Graphs (**b**–**d**) show metal content in ppm for additive metals (left), fouling metals (middle), and wear metals (right). If no bar is visible, the value of the related parameter is zero.

The same experiment was repeated with ULO after 1st and 2nd distillation. Each time, new fresh membranes were used. The flux was again stable and measured to be 8.8 and 9.8 kg/h·m^2^ respectively, very similar to that of the untreated ULO. Figure 7 presents the retentions observed for the various parameters, highlighting that only the relatively low metal retentions change following pre-treatment. Indeed, for metals that were not retained near 100%, retention rates increased with more pre-treatment. However, for S, Si, and Pb, retention never exceeded 90%.

**Figure 8:**
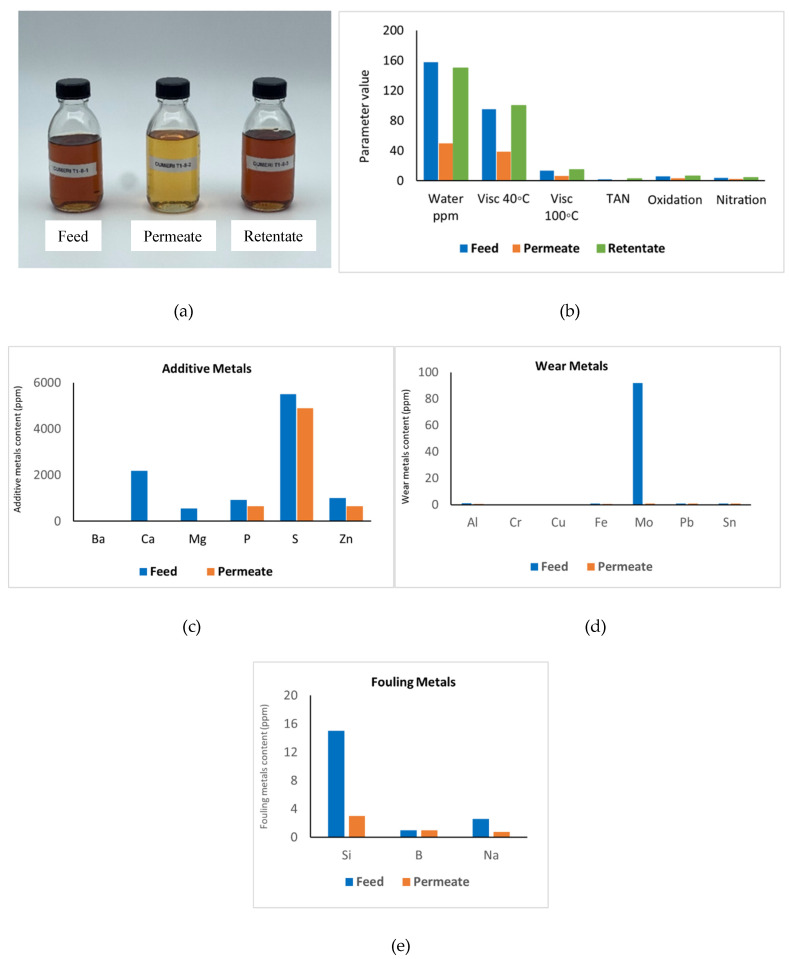
Clean commercial LO before and after UF treatment using a 10 nm native membrane. (**a**) The picture shows the feed, permeate, and retentate from left to right. Graph (**b**) shows values for general characteristics (parameter on the *x*-axis, water content in ppm, kinetic viscosity at 40 °C and/or 100 °C in cStokes, total acidity number TAN in mg KOH/g, oxidation and nitration level in Abs/0.1 cm, and diesel and naphtha in %). Graphs (**c**–**e**) show metal content in ppm for additive metals (left), fouling metals (middle), and wear metals (right). If no bar is visible, the value of the related parameter is zero.

For comparison, we also measured a new 10 nm native benchmark membrane in clean commercial LO. The flux was again stable and now 5 kg/h·m^2^ compared to the 7 to 9 kg/h·m^2^ for as-received ULO. This flux decline may be due to the higher viscosity of the clean LO but may also be influenced by the different composition (see Table 3 and Figure 8). The data presented in Figure 8 outlines the permeate quality. Based on the analysis, it was concluded that water, viscosity, acidity, oxidation, nitration, and many metals were all effectively reduced, with retention levels very similar to the ones observed for (pre-treated) ULO. As for ULO, metals such as P, Zn, Fe, S and B showed lower removal efficiency. In this case, the feed and retentate had a brown, oily color, while the permeate had a visibly brighter color. The black color observed in all ULO samples most likely originated from the high amount of oxidation and nitration components due to LO use.

### 3.2. Flux Performance Using Clean Base Oil

To get more understanding of the flux values measured in ULO and LO streams, filtration was also performed in our standard conditions using clean base oil without additives or impurities as a feed. For this purpose, the purified product of Osilub, OSIL150, was utilized. As mentioned in Section 2.2, this is not 100% clean base oil, as some sulfur impurities are still in there. Not only were native 10 nm benchmark membranes used in this study but also other native membranes with smaller and bigger pore sizes and hydrophobic versions of some of these membranes, created by HOC 2% grafting.

**Figure 9:**
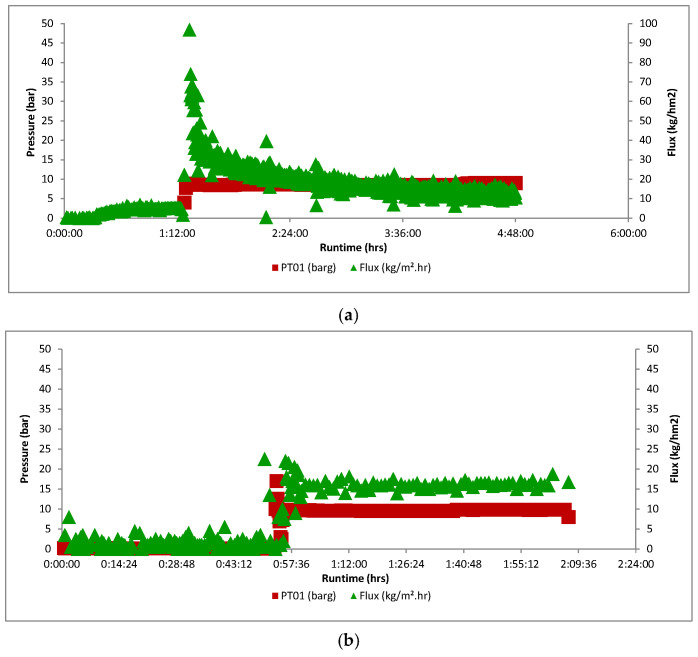
(**a**) Flux evolution of a 10 nm native membrane and (**b**) 10 nm HOC 2% membrane using OSIL150 as feed.

Figure 9a shows the flux evolution of an experiment with a native 10 nm benchmark membrane. The flux is clearly not stable but drops from a level around 70 kg/h·m^2^ to a +/− stable value of 12 kg/h·m^2^. For a 10 nm HOC 2% membrane, on the contrary, the flux is immediately stable at a value of about 16 kg/h·m^2^ (Figure 9b). The reason for the flux drop of the native membrane is unclear but might be related to the hydrophilic character of the native membrane. An elucidation of the mechanisms behind the difference is very complex and was beyond the scope of this study. The complexity of flux decline for native membranes in hydrophobic matrices has been observed before, e.g., in pure hexane flux studies [49], highlighting the significant influence of membrane surface chemistry. In Table 4, all flux values are summarized. In the case of a flux drop, the starting value and +/− stable value are mentioned.

When we look at the stable values of the different membranes, it becomes clear that flux values for native and hydrophobic membranes with the same pore size (before grafting) are very similar, especially for the 10 and 30 nm membranes. For the tighter 5 nm membranes, the effect of the chemistry seems to be larger, consistent with the stronger interactions expected between oily feed and pore walls in the smaller pores. Moreover, the flux values increase strongly with pore size and evolve with the square of the pore size as expected from the Hagen–Poiseuille equation. For the 10 nm native membranes, the flux of 12 to 16 kg/h·m^2^ is in the same order of magnitude as the ULO flux of 7 to 9 kg/h·m^2^. This means that the influence of the concentration/cake layer is not extreme for this membrane. It will be shown in Section 3.3 that the difference between ULO flux and OSIL150 flux is much larger for 30 nm membranes, which might be due to partial pore blocking of the retained impurities for these bigger pores. In the future, a second paper will indeed show that 30 nm membranes show much higher irreversible fouling than 10 nm membranes.

### 3.3. Effect of Membrane Grafting on Performance in ULO

A large number of different membranes with varying grafting methods were examined with ULO at the same operating conditions of 10 bar, 2 m/s and 110 °C. The membranes varied in terms of pore size and material. The type of support material used was primarily alumina, but a full titania membrane was also used (see Section 2.1). The top-layer material varied between TiO_2_, ZrO_2_, and Al_2_O_3_. A large range of surface chemistries were also created by grafting, including native hydrophilic, amphiphilic, hydrophobic, oleophilic and extra hydrophilic properties. The surface chemistry modification was achieved through different grafting techniques, which included varying grafting coverage and modifications limited to the top layers or extended to the support material, as explained in Section 2.1.

**Figure 10:**
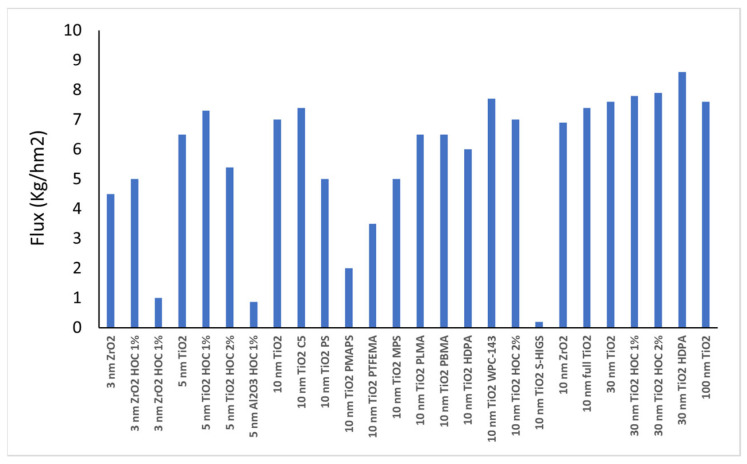
Fluxes in (kg/h·m^2^) observed for different UF membranes.

**Figure 11:**
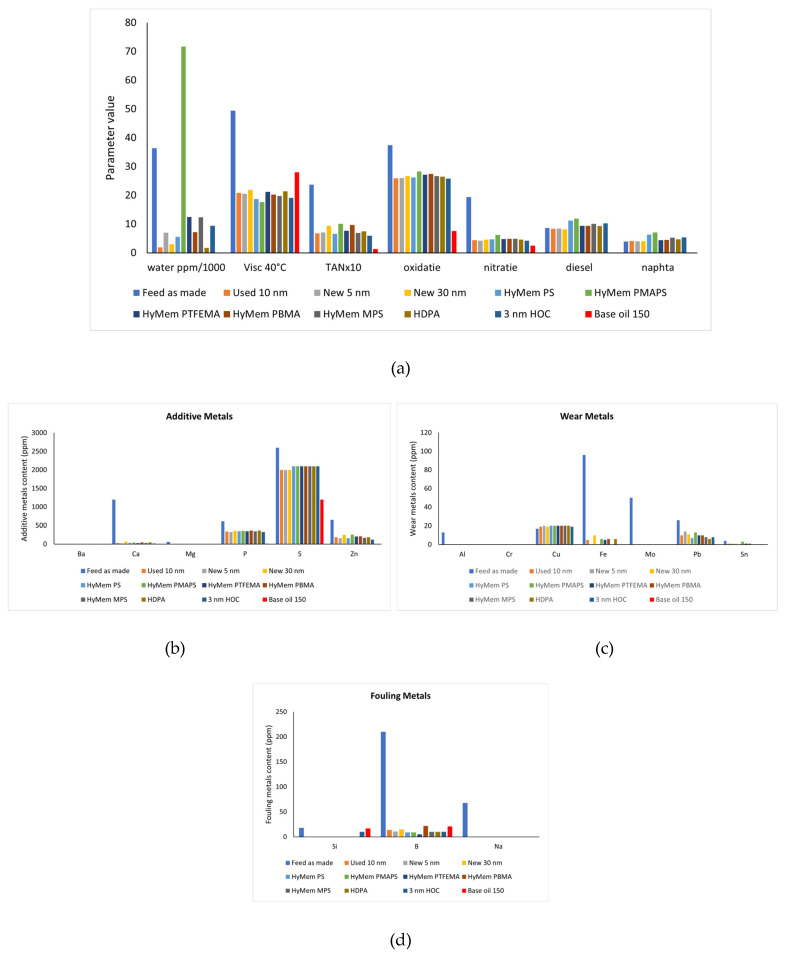
Performance of different UF membranes tested with the same batch of ULO. The feed composition is shown in blue and the OSIL150 purified product in red for comparison. Graph (**a**) shows values for general characteristics (parameter on the *x*-axis, water content in ppm, kinetic viscosity at 40 °C and/or 100 °C in cStokes, total acidity number TAN in mg KOH/g, oxidation and nitration level in Abs/0.1 cm, and diesel and naphtha in %). Graphs (**b**–**d**) show metal content in ppm for additive metals, fouling metals, and wear metals. If no bar is visible, the value of the related parameter is zero.

A first set of native and grafted membranes was used to filter the same batch of ULO. Figure 10 and Figure 11 give an overview of the performances. The data shows that the performances are remarkably similar for all membranes, closely resembling the performance of the reference native 10 nm membrane. However, there are two exceptions: the water retention and the flux. Indeed, as can be observed from Figure 11, the viscosity, TAN, oxidation and nitration level, and diesel and naphtha content, as well as full metal content, in the permeate are very similar for all membranes. Do note that most membranes tested in this first series do show water removal. However, one membrane (10 nm TiO_2_ PMAPS) shows strong negative retention for water, meaning that the water content in the permeate is much higher than in the feed. This is also confirmed by the very light, transparent color of the permeate containing about 80% water (as measured by Karl Fisher). In this strongly grafted (densified), hydrophobic membrane, the oily matrix is unable to pass through, allowing only small, free water molecules (and most likely naphtha molecules) to move across the membrane. Notably, the retention for all other parameters (e.g., metal retentions) was similar to that observed in the high-flux and high-water-retention membranes.

In the same way, we performed ULO filtration with all other membranes mentioned in Section 2.1. The new results confirm consistent performance for all membranes, except for flux and water retention. Table 5 summarizes the fluxes and water retentions of all membranes tested (when two flux values are mentioned, these are measured with different ULO batches). Overall flux values range from very low (0.2 kg/h·m^2^ ) up to 9 kg/h·m^2^, while water retention varies from negative (clear permeate) to almost 100%. Notably, flux values of open 30 and 100 nm membranes do not rise above the value measured for 10 nm membranes, and this might be due to partial pore blocking of the retained impurities in these bigger pores. From the table, a correlation is noticeable between flux and water retention: membranes with the highest fluxes also exhibit the highest water retentions and vice versa. Peculiar behavior was again observed in relatively tight membranes with high hydrophobicity. These membranes exhibited particularly low fluxes, near or below 1 kg/h·m^2^, along with a transparent permeate with a high water content as confirmed by Karl Fischer. As a result, these membranes are unsuitable for use as ultrafiltration (UF) membranes for ULO purification.

**Figure 12:**
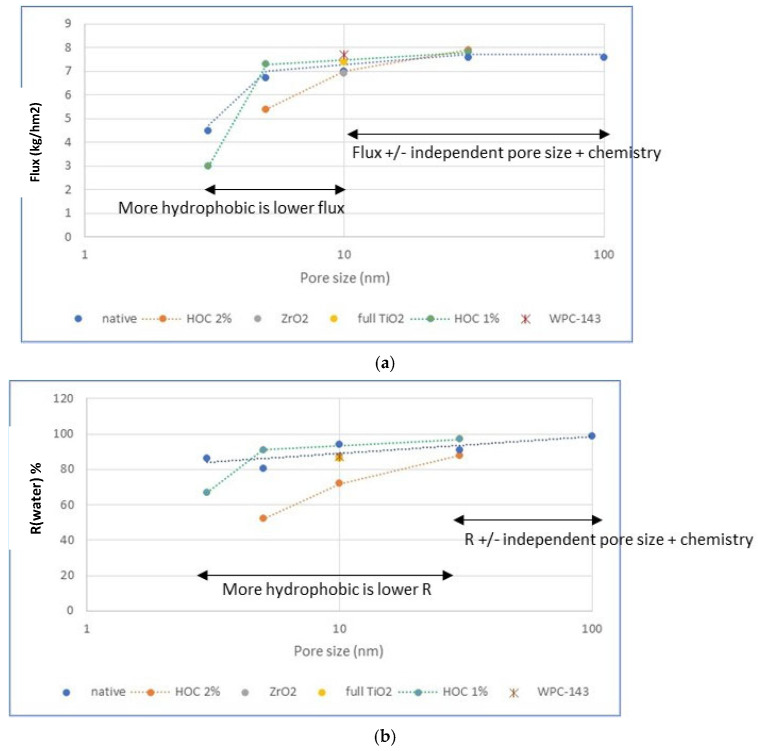
Fluxes and water retention as functions of pore size before grafting and membrane chemistry (**a**) Flux as a function of pore size for diffrent membrane chemistries, (**b**), Water retention as a function of pore size for diffrent membrane chemistries.

To better understand the impact of membrane chemistry on performance, Figure 12 illustrates the relationship between pore size of the membranes before grafting (*x*-axis) and the resulting flux and water retention values (*y*-axis) for various membrane chemistries. In these graphs, we included the native membranes and the membranes grafted with HOC 1% and 2%, as this type of grafting was used on various pore sizes. None of the Si-ATRP membranes are included (except for the WPC-143 membrane), as this type of grafting was mainly applied on 10 nm membranes. Figure 12 shows that, beyond a certain threshold of pore size, the performance of the membranes reaches a maximum and becomes independent of the specific chemistry. The threshold pore size (before grafting) is lower for fluxes (10 nm) than for water retention (30 nm). For pore sizes below the threshold, fluxes and water retention decrease with decreasing pore size (before grafting), and chemistry starts to have an influence. Comparing HOC 1%- and HOC 2%-grafted membranes, it can be concluded that more strongly grafted membranes show a stronger decline in flux and water retention. This fits the fact that stronger grafting typically leads to higher surface coverage and therefore lower effective pore size of the grafted membrane (stronger densification). Looking at the results of all 10 nm membranes in Figure 12 and Table 5, it can also be observed that the top-layer material (ZrO_2_ or TiO_2_) or support material (Al_2_O_3_ or TiO_2_) does not have an influence on flux or water retention. These observations are key in narrowing down the membrane selection for ULO treatment.

## 4. Conclusions

This paper focused on studying ceramic UF membranes with different pore sizes (3 nm, 5 nm, 10 nm, 30 nm, and 100 nm) and different surface chemistries for the recovery of ULO at high temperature. Surface chemistry was altered by using one of four grafting technologies and applying a variety of functional groupt. This allowed us to alter the typical hydrophilic nature of ceramic membranes to be more hydrophobic and/or oleophobic, with a possible positive effect on flux and/or performance in the oily feed streams. In total, 35 membranes were included in the research. The native 10 nm TiO_2_ membranes were considered as benchmark membranes.

The oily feed streams used for the UF experiments were as-received ULO from partner company and French re-refiner Osilub and ULO after the 1st and 2nd distillation steps performed at Osilub, intended to remove water, and naphtha and diesel respectively. For comparison, clean commercial lubricant oil was also used, as well as one of the purified products of Osilub, OSIL150, which is very close to clean base oil.

For ULO filtration, the native 10 nm benchmark membranes show a stable flux of 7 to 9 kg/h·m^2^ at 110 °C. The relatively low value of the flux is influenced by the high, temperature-dependent viscosity of the feed but also by the formation of a concentration polarization or cake layer. Permeate analysis proves that UF offers good first purification of ULO. The results show an unexpected high water retention of over 90%, most likely due to the stabilization of the water molecules with surfactant-like additives or other impurities in the ULO. Moreover, high reductions in viscosity, TAN, oxidation, and nitration component levels are reached. Metal content, arising from lubricant additives, fouling and wear, is also highly decreased. Several metals show retentions near 100%; however, retentions for P, S, Zn, Si, Cu, Fe, and Pb are lower and do not allow the quality of the Osilub products to be reached by UF alone. As expected, UF does not remove any diesel or naphtha. Experiments with ULO pre-treated with one or two distillations show similarly good results and even somewhat increased retentions for the metals that are not 100% removed. UF on clean commercial lubricant oil shows lower fluxes due to higher feed viscosity and a different composition, but retention levels are consistent with the results on the other oily feeds.

Clean base-oil flux results on native and hydrophobic membranes with varying pore size show unclarified flux decline in many cases, especially for native membranes. Stabilized flux values vary with the square of the pore size, consistent with Hagen–Poiseuille, and are quite independent of surface chemistry. For 10 nm membranes, the base-oil flux values are also in the same order of magnitude as the ULO fluxes. For membranes with a larger pore size, the differences are much higher, most likely pointing to partial pore blocking by retained impurities.

Subsequently, different membranes with varying pore sizes and grafting were tested in ULO. Fluxes never exceeded the ones for the 10 nm benchmark membranes, elucidating that surface chemistry does not help to improve the flux. In general, it was observed that the performance of all membranes was very similar, except for their flux and water retention. Moreover, a correlation was observed between flux and water retention: the higher the flux, the higher the water retention, and vice versa. The very-low-flux membranes even show transparent permeates containing high amounts of water and thus negative water retention. This suggests that mainly small, free water and naphtha molecules pass through these tight membranes, but the oily matrix cannot. Plotting fluxes and water retention as functions of membrane pore size before grafting allowed us to conclude that membrane chemistry only influences membrane performance when the effective pore size (after grafting) is comparatively small.

The observations allow us to make an educated selection of the best ceramic UF membranes for ULO treatment at high temperature.

## Figures and Tables

**Table 1:** Overview of the different native membranes used in this study. The membrane type was defined by the pore size and material of the top layer.

Membrane Type	Support Material	MWCO (kDa)
3 nm ZrO_2_	Al_2_O_3_	2
5 nm TiO_2_		8.5
5 nm Al_2_O_3_		5
10 nm TiO_2_		20
10 nm ZrO_2_		20
30 nm TiO_2_		100
100 nm TiO_2_		-
10 nm TiO_2_	TiO_2_	20

**Table 2:** Overview of the ceramic UF membranes with different grafting used for used lubricant oil purification in this study.

Support	Grafted Group (Short)	Grafted Group (Full)	Grafting Method
3 nm ZrO_2_	no HOC 1%	no hydrophobic hydrocarbon	no SI SI
	HOC 2%	hydrophobic hydrocarbon	
5 nm TiO_2_	no	no	no
	C8	octyl	Grignard
5 nm TiO_2_	HOC 1% HOC 2%	hydrophobic hydrocarbon hydrophobic hydrocarbon	SI SI
5 nm Al_2_O_3_	no HOC 1%	no hydrophobic hydrocarbon	no SI
10 nm TiO_2_	no	no	no
	C1	methyl	Grignard
	Ph	phenyl	Grignard
	C5	pentyl	Grignard
	C8	octyl	Grignard
	C12	dodecyl	Grignard
	C18	octadecyl	Grignard
10 nm TiO_2_	PS	polystyrene	Si-ATRP
	PMAPS	3-methacryloxypropylmethyldimethoxysilane	Si-ATRP
	PTFEMA	2,2,2-trifluoroethyl methacrylate	Si-ATRP
	MPS	α-methylstyrene	Si-ATRP
	PLMA	lauryl methacrylate	Si-ATRP
	PBMA	n-butyl methacrylate	Si-ATRP
	PDMS	polydimethylsiloxane	Si-ATRP
10 nm TiO_2_			
	PPA	phenyl	PA
	HDPA	hexadecyl	PA
10 nm TiO_2_	WPC-143	nanoparticle coating lowering roughness	Si-ATRP
10 nm TiO_2_	HOC 2% S-HIGS	hydrophobic hydrocarbon hydrophilic hydrocarbon	SI SI
10 nm full TiO_2_	no	no	no
10 nm ZrO_2_	no	no	no
30 nm TiO_2_	no	no	no
	HDPA	hexadecyl	PA
30 nm TiO_2_	HOC 1% HOC 2%	hydrophobic hydrocarbon hydrophobic hydrocarbon	SI SI
100 nm TiO_2_	no	no	no

**Table 3:** Characteristics of the different ULOs.

Feed Streams	Water (ppm)	Visc. at 40 °C (cSt)	Visc. at 100 °C (cSt)	TAN (mg KOH/g)	Oxidation (Abs/0.1 mm)	Nitration (Abs/0.1 mm)	Diesel %	Naphtha %
ULO without neutralization	63,000	53	8.7	1.96	29	24.4	5.8–7	1–6.8
ULO 1st dist	870	56.1	9.9	0.83	20.9	11.3	4.5–5.5	0.5
ULO 2nd dist	119	58.4	9.9	0.59	19.9	8.5	-	-
OSIL150	20	28.4	5.3	0.07	7.6	2.5	-	-
Commercial unused LU	158	95.5	13.8	1.91	5.9	4.1	-	-

**Table 4:** Flux values observed for different native and HOC 2%-grafted membranes.

Pore Size	Flux Native Membranes	Flux HOC 2% Membranes
5 nm 10 nm	20 to 4 kg/h·m^2^ 70 to 12 kg/h·m^2^	9 kg/h·m^2^ 16 kg/h·m^2^
30 nm	300 to 240 kg/h·m^2^	300 to 200 kg/h·m^2^

**Table 5:** Fluxes and water retentions for all UF membranes tested.

Support	Grafted Group Short	Grafted Group Full	Flux	R (Water)
3 nm ZrO_2_	no	no	4.5	86
	HOC 1%	hydrophobic hydrocarbon	5.0	67
	HOC 1%	hydrophobic hydrocarbon	1.0	clear permeate
5 nm TiO_2_	no	no	6.9/6.5	81
	HOC 1%	hydrophobic hydrocarbon	7.3	91
	HOC 2%	hydrophobic hydrocarbon	5.4	52
5 nm Al_2_O_3_	HOC 1%	hydrophobic hydrocarbon	0.25/1.5	clear permeate
10 nm TiO_2_	no	no	7.0	94
10 nm TiO_2_	C5	pentyl	7.4	93
10 nm TiO_2_	PS	polystyrene	5.0	58
	PMAPS	3-methacryloxypropylmethyldimethoxysilane	1.0/3.0	clear permeate
	PTFEMA	2,2,2-trifluoroethyl methacrylate	3.5	63
	MPS	α-methylstyrene	5.0	53
	PLMA	lauryl methacrylate	6.5	91
	PBMA	n-butyl methacrylate	6.5	69
	HDPA	hexadecyl phosphonic acid	6.0	94
10 nm TiO_2_	WPC-143	lowering roughness	7.7	87
10 nm TiO_2_	HOC 2%	hydrophobic hydrocarbon	7.0/7.0	72
	S-HIGS	hydrophilic hydrocarbon	0.2	clear permeate
10 nm ZrO_2_	no	no	6.9	87
10 nm full TiO_2_	no	no	7.4	86
30 nm TiO_2_	no	no	7.6	91
	HOC 1%	hydrophobic hydrocarbon	7.8	97
	HOC 2%	hydrophobic hydrocarbon	7.9	88
30 nm TiO_2_	HDPA	hexadecyl phosphonic acid	8.6	95
100 nm TiO_2_	no	no	7.6	99

## Data Availability

The raw data supporting the conclusions of this article will be made available by the authors on request.
